# Co-producing shared wound care education: experiences of service users and healthcare professionals

**DOI:** 10.1080/17482631.2026.2637799

**Published:** 2026-03-02

**Authors:** Joanna Blackburn

**Affiliations:** a School of Human and Health Sciences, University of Huddersfield, Queensgate, Huddersfield, West Yorkshire, England, UK

**Keywords:** Co-production, shared wound care, patient education, qualitative research, thematic analysis, person-centred care, community nursing

## Abstract

**Purpose:**

Shared wound care promotes collaboration between patients and healthcare professionals (HCPs) to improve wound management outcomes and enhance patient autonomy. Educational resources to support shared wound care are typically developed without the direct involvement of service users or HCPs, limiting their relevance, accessibility, and impact in community practice. This study aimed to co-produce an educational resource to support shared wound care in community settings by integrating the perspectives of both service users and HCPs.

**Methods:**

A four-phase explanatory sequential mixed-methods design was employed involving an online survey, focus groups and semi-structured interviews to explore the experiences, needs and preferences of HCPs and service users. Semantic thematic analysis was used to analyse the data.

**Results:**

Both groups prioritised clear information in a leaflet format about wound infection, wound dressing changes and the normal wound healing process to facilitate involvement in shared care tasks. Co-designing the educational resource ensured it was both clinically and contextually relevant, reflecting the real-world experiences of patients and clinicians.

**Conclusions:**

Co-producing educational resources enhances their accessibility and applicability and supports continued engagement from service users in their own care, providing a model for collaborative resource development that aligns with person-centred and self-management principles in community healthcare.

**Patient or public contribution:**

Service users with lived experience were involved in the conduct of this study.

## Introduction

Effective wound management is complex and requires a multidisciplinary approach, combining clinical care with addressing the psychosocial and environmental challenges of living with a wound. These principles align with the person-centred care approach, which considers the patients' preferences, values, and support networks in treatment decisions (Allen, 2014). Actively involving patients in their own healthcare is a recognised approach and the collaboration between patients and healthcare professionals (HCPs) in treatment decisions improves patient adherence to treatment plans and satisfaction with care for patients suffering from long term conditions including diabetes (Chen et al., 2018), chronic kidney disease (Green et al., 2024) and rheumatoid arthritis (Bhangu et al., 2023).

Defined by the Self Care Forum (2020) as “*the actions that individuals take for themselves, on behalf of and with others in order to develop, protect, maintain and improve their health, wellbeing or wellness”,* self-care exists on a continuum ranging from complete self-care to full medical care. The approach involves varying levels of contribution from patients, caregivers and HCPs, with the balance being determined by the patients extent of involvement. Shared care refers to a process that involves patients, their caregivers, and HCPs working collaboratively for treatment and care planning with patients undertaking a variety of condition-specific tasks to optimise health and lifestyle outcomes (National Institute for Health and Care Excellence (NICE), 2024) dependent on the patients' individual needs and expectations (Sutherland & Levesque, 2020). NHS England's (2019) guidance on involving people in their own care outlines several benefits to this model, including improved health and wellbeing, improved experiences of care and reduced financial burden.

### Shared care in wound management

Wound care cost the UK healthcare system an estimated £8.3 billion between 2017 and 2018; £5.6 billion of these costs were related to managing unhealed wounds in the community (81%) and were associated with over 54 million community nurse visits (Guest et al., 2020). According to Moore et al. (2022), a shared care approach in wound management could result in 3.5 billion hours of nursing time saved globally by 2030, and therefore provides a sustainable opportunity for wound care. The National Wound Care Strategy Programme (NWCSP, 2020) guidelines offer recommendations for implementing shared wound care which include supporting patient confidence through education. NICE (2024) guidelines also support shared care for chronic wounds, such as venous leg ulcers (VLUs). Shared wound care can involve a plethora of different tasks including wound cleaning, wound dressing and education to support the recognition of possible infection (Kapp & Santamaria, 2017; Žulec et al. 2019). Incorporating shared care into treatment protocols has been found to reduce healing times, nursing time associated with treatment and care, and limit financial burden (Lindholm & Searle, 2016; Clemett et al., 2024). Furthermore, adherence to treatments such as compression or offloading therapy is associated with lower recurrence rates of VLUs, while non-adherence has been found to increase recurrence risk (Armstrong & Meyr, 2019).

### Challenges to shared wound care

Whilst the shared care model offers notable benefits for wound management, its implementation faces several challenges, and many individuals living with wounds report insufficient support to achieve greater independence (Blackburn et al., 2021). Living with a chronic or non-healing wounds can negatively affect psychological well-being and reduce motivation, impeding recovery (Redmond et al., 2025), and a lack of wound care education can increase levels of anxiety (Blackburn & Ousey, 2023). This is important, as adherence to treatment is evidenced when patients are informed about their care (Moffatt et al., 2017) and being involved in post-discharge wound care decisions result in patients being significantly more likely to feel competent managing their wounds at home (Tobiano et al., 2023). However, Meijers et al. (2019) identified no significant improvement in treatment adherence confidence among patients with diabetic foot ulcers following interventions aimed at enhancing shared decision-making, suggesting that patients may benefit more from interventions aimed at increasing their motivation.

Individuals with wounds may experience symptoms such as pain and limited mobility, which can affect independence and social participation (Blome et al., 2014; von Stülpnagel et al., 2021), impacting engagement in shared care (Olsson et al., 2019). Non-adherence to treatment is important to understand, with factors such as pain and discomfort, limited knowledge, mobility impairments, and restricted financial resources being cited in the literature (Chitambira, 2019). Pain and discomfort (Finlayson et al., 2014; Kapp & Miller, 2015) and insufficient understanding of the importance and effectiveness of treatment adherence are frequently cited barriers (Van Hecke et al., 2009; Weller et al., 2016) and suggest that interventions need to focus on a holistic multidimensional approach incorporating effective pain management and tailored, ongoing patient education. The World Health Organisation (WHO) (2016) guiding principles for integrated care state patients and their caregivers should be engaged in decision making in wound management to maximise a positive impact on their physical, psychological, and social needs.

Active patient participation in healthcare relies on health literacy and (Self Care Forum and Self Care Academic Research Unit (SCARU), 2024) patients generally report better experiences when shared decision making in wound care is linked to goal setting, reducing conflict and improving knowledge (Žulec et al., 2019; Clemett et al., 2024). Improved quality of life is also evidence through reducing the need for frequent hospital visits for treatment and care (Goh & Zhu, 2024). However, Meijers et al. (2019) found that healthcare providers still make most treatment decisions, with patient involvement limited due to barriers such as inadequate clinician training, assumptions about health literacy, and time constraints. A recent study (Blackburn & Ousey, 2023) exploring the experience of shared wound care during a pandemic found that most patients and their carers undertook shared care activities in the absence of any specific knowledge or education. The participants advocated for the development of general and specific wound care education to support shared care, focusing on specific aspects to enable them to be more actively involved. HCPs in this study were unclear how best support their patients and felt they lacked the guidance to effectively encourage their patients to undertake wound care tasks.

### Limitations of current shared care models

Shared care education often lacks collaboration with service users and HCPs. Co-production, as defined by NHS England, involves equal partnerships and early engagement in service design between professionals and service users in the design, delivery, and evaluation of services (Makey et al., 2022). Although shared wound care is increasingly recognised as a means to enhance patient engagement and optimise care delivery, current models of wound care education are frequently developed without meaningful collaboration between service users and service delivery. This limits the relevance, accessibility, and sustainability of educational interventions, leading to variability in patient understanding, inconsistent application of shared care principles, and reduced engagement. Wound care resources and educational pathways often fail to include patient or clinician input during their design (Blackburn & Ousey, 2023) and whilst patient participation and self-care are encouraged within chronic wound management, existing frameworks provide limited guidance on how to effectively support such involvement (Smith & Sharp, 2023). Broader evidence on co-production within health systems indicates that service design commonly lacks partnerships with service users, despite policy recommendations for participatory approaches (Conquer, 2025). To address this, the present study aimed to co-produce an educational resource to support shared wound care in community settings.

## Aim

The aim of this study was to co-design an educational resource to support shared wound care for patients living with a wound in the community setting.

## Ethical approval

Ethical approval was obtained from the School Research Ethics and Integrity Committee (SREIC) at The University of [insert name here] (reference number: SREIC/2023/086). The study was conducted in accordance with the ethical principles of the Declaration of Helsinki (World Medical Association, 2013).

## Methods

**Figure 1.**
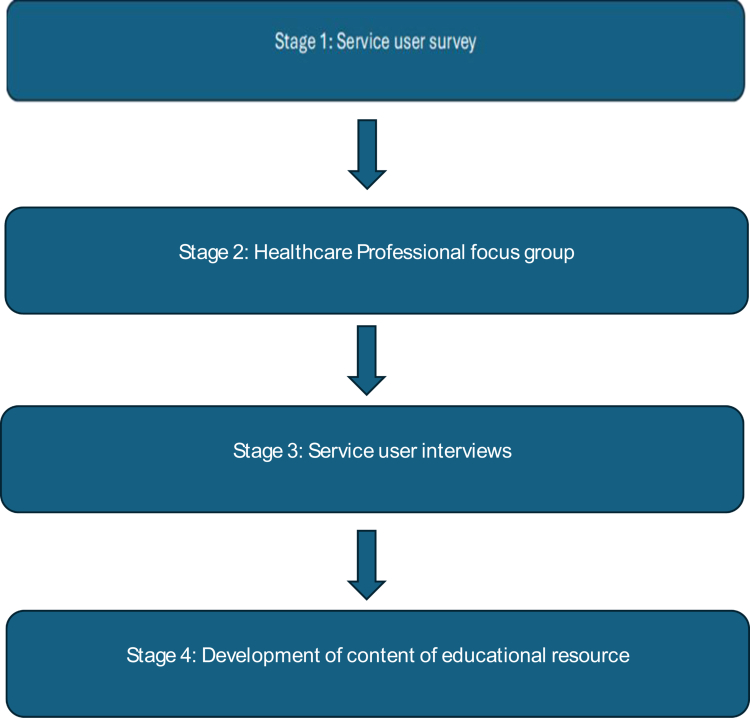
Methodological steps of the research.

An explanatory sequential mixed-methods design was used to develop the education with service users and HCPs. The study incorporated four main phases that were designed to run sequentially; each informing the next to gradually develop the educational resource (Figure 1). All activities were conducted virtually and facilitated by the lead researcher. The four phases were as follows:

1. Design and dissemination of a service user survey.
2. HCP focus group.
3. Individual qualitative semi-structured interviews with service users.
4. Educational resource development.

## Participants and recruitment

### Service user group

A service user group (*n* = 6) was established to guide the development and co-design of the educational resource and consisted of individuals with lived experience of wounds, who were experts by experience (3 male, 3 female; age range 45–78 years). Participants included those with experience of surgical, chronic and necrotic wounds managed within community and outpatient settings. Surgical wounds comprised primarily orthopaedic and surgical wound dehiscence. Chronic wounds included venous leg ulcers and diabetic foot ulcers. This population reflects the typical case mix encountered in community wound care, encompassing both post-surgical management and long-term chronic wound maintenance. The participants had engaged in a previous study exploring shared wound care during a pandemic [insert reference of authors here] and had expressed interest in contributing to future research activities. Participants were considered representative of the larger population of interest. Participants were provided with an information sheet via email informing them about the study and written informed consent was obtained prior to undertaking any research activities.

### Healthcare professionals (HCPs)

A purposeful sample of HCPs (*n* = 6) were recruited through the research teams' existing connections with Tissue Viability Services across the United Kingdom (UK) and the Tissue Viability Facebook group page. Participants were HCPs working in wound care in the UK with experience of shared care in community settings ranging from community nurse to matron. Participants were provided with an information sheet via email informing them about the study and written informed consent was obtained prior to undertaking any research activities.

## Phase one: service user survey

The first phase of the project was designed to obtain baseline information on the service user needs and preferences for an educational wound care resource. An online survey consisting of 15 open-and-closed questions was developed using the Qualtrics platform and distributed to service users using an email link in March 2024. Questions explored previous experiences of shared wound care education and beliefs about the needs and requirements for an educational resource to support shared wound care. The survey was structured into three main sections: (1) service user demographics and wound care background, (2) previous experiences of shared wound care education, and (3) preferences for the format, accessibility, and content of a future educational resource. The first section collected information on age, gender, wound type and frequency of dressing changes, providing context on participants' experiences with wound management. The second section included both closed- and open-ended questions exploring users' previous experiences of wound care education, their level of participation in shared care, and perceived barriers or enablers to engagement. The final section focused on identifying preferred learning formats (e.g. printed materials, videos, online platforms), content priorities (e.g. infection signs, dressing changes, nutrition), and accessibility needs, including visual or literacy considerations. The survey was reviewed by two wound care specialists to ensure relevance of the questions. Closed-ended responses were analysed using descriptive statistics to identify common trends, while open-ended responses were analysed using thematic analysis to capture service user perspectives and inform the design of a user-centred educational resource.

## Results

Education on the signs and symptoms of infection, types, forms and functions of wound dressings and dressing changes, and information on wound types and the normal wound healing trajectory, were identified as the most important topics to include in an educational resource for shared wound care. Pictures and diagrams, demonstrating wound care techniques, were also favoured and presenting the information in a leaflet format, provided by a nurse during a home visit, were felt to be the best way of receiving education.

These findings were used to inform the HCP focus group in phase two.

## Phase two: HCPs focus group

An online focus group was undertaken with HCPs working in wound care (*n* = 6) in the community setting, to explore their perspectives on shared wound care education. The focus group was facilitated by a topic guide using findings from the literature, the research teams' previous research, and the findings from the service user survey in phase one. Participants were asked to discuss the type and format of education they thought would be useful to include in a wound care resource to support shared wound care. Findings from phase one were used to support the discussion.

## Data recording and analysis

The focus group was audio and video recorded with the participant's consent via Microsoft Teams to enable transcription and analysis. The recording was transferred to an encrypted storage device and identified with a code, transcribed verbatim by University employed transcribers and anonymised to remove any information, which might identify the respondents. Transcripts were entered into NVIVO 15 qualitative data analysis software to aid data management analysis and retrieval. Semantic thematic analysis (Braun & Clarke, 2006, 2021) was used to analyse the data. This approach is widely used in qualitative data analysis, focusing on the description and semantic content of the participants responses. The approach aims to identify recurring themes based on the explicit and surface-level meanings in the data rather than seeking to interpret underlying meanings. Broad themes based on the participants responses were identified from the dataset to generate an overall view of the HCPs perspectives on an educational resource to support shared wound care.

## Themes

Three themes were identified from the dataset that described the HCPs perspectives on shared wound care education. These were (1) wound dressings and wound infection, (2) skin care and lifestyle, (3) resource format, (4) format and accessibility.

### Theme 1: wound dressings and wound infection

Education on wound dressings and recognising signs and symptoms of infection were considered key areas of focus to enable shared wound care. Participants felt that patients changed their dressings too frequently and emphasised the need for education on the normal wound healing process and how wound dressings facilitate this. Providing clear information on what a wound might look like when a dressing is removed was also seen as essential to build confidence.

*“I think people change their dressings far too much really. I think they think that they need to put a clean dressing on every day when really a lot of the dressings are designed to stay on for seven days. So it's a bit of education around that about what's happening to the wound while you've got the dressing on. It might not look, sometimes it looks worse before it starts to get better and I don't think they understand that.”* (District nurse)

HCPs recommended guidance on the differences between inflammation and infection, how wound infections can be treated with dressings, and basic information on antimicrobial resistance (AMR) and sepsis. These were considered important aspects of shared care that would support knowledge building around understanding the scientific principles of wound healing.

“*It would be good to have something that's really clear about that, the difference between inflammation and infection, it might not look perfect to you, but this is when we want you to ring us and pictures would be helpful.”* (Senior Nurse)

*“Well they could say that wound infections can be treated by the dressings themselves and therefore antibiotics are not always necessary.”* (Tissue Viability Nurse)

### Theme 2: skin care and lifestyle

Key topics included routine skin care (such as moisturising), bathing and cleaning advice, and lifestyle factors such as smoking, nutrition, and medication that affect wound healing. Education about these factors was seen as crucial for encouraging effective wound care and promoting wound healing.

*“You could just put something very brief, risk factors or things that can help is reduce smoking, having a good diet, some medications might interfere –speak to your professional about that, just keep it simple.HCPs recommended guidance”* (Podiatrist)

### Theme 3: resource format

Distribution of education using a leaflet format was identified as a suitable method for reaching a broad range of service users, utilising simplified language and images to support understanding. Real life and cartoon pictures were thought to be useful to aid understanding and ensure inclusivity.

A website was also thought as being beneficial for offering more comprehensive information, such as educational videos and demonstrations of HCPs performing basic wound care tasks (including opening a dressing pack, cleaning a wound, and applying a dressing). HCPs thought that a question and answers or a frequently asked questions (FAQs) section could be included in either a leaflet or on a website and it was suggested that this should include information relating to dressing changes wound healing, wound impact of medication on wound healing and nutrition and exercise.

## Phase three: service user interviews

Individual qualitative semi-structured interviews were undertaken with service users (*n* = 6) to explore their views on the content, format and accessibility of the educational resource in more detail. They were asked to describe their thoughts on the findings from phases 1−2 and discuss the type of education and information that would enable them, and others, to engage in shared care successfully.

## Data recording and analysis

The interviews were audio recorded and transcribed as in phase 2 and analysed using semantic thematic analysis (Braun & Clarke, 2006; Braun & Clarke, 2021). Three themes were identified that described the service user views on shared wound care education. These were (1) wound dressings and wound healing, (2) infection and inflammation, and (3) skin care advice and healthy lifestyle behaviours.

## Results

### Theme 1: wound dressings and wound healing

Service users described the importance of including education on wound dressings, wound dressing changes, and how a dressing supports wound healing in an educational resource, reinforcing the HCP views. This was important, as several misconceptions were identified from the interviews, including believing that a wound dressing should be changed daily and a belief that seeing strikethrough on a dressing was an indication it should be changed. Almost all service users thought that a wound should be exposed to the air to promote healing and that wound dressings cause the wound to “sweat” and delay healing. This also supported the HCPs suggestion of providing education on how a wound dressing may look throughout the different stages of healing. Providing education on the scientific properties of how a wound dressing promotes healing, to enable patients to follow the correct guidance was also essential.

*“I have been wearing a wrap which is an elasticated piece of equipment that wraps around your leg and the purpose of that is to force the blood back up the leg from the foot and again I was only told this last week, that that was the one thing that would heal the wound. It's not, the dressing helps, but it's the wrap, the elasticated wrap which you've got to wear all day, every day and that is the one thing which does assist in healing the wound.” (Participant with a chronic wound)*

### Theme 2: infection and inflammation

Consistent with the findings from the HCP focus group, participants thought that including explanations about the differences between normal wound healing, infection, and inflammation, as well as information the basic signs and symptoms of infection, should be included as part of shared care education. It was also important to include information on what a normal healing wound should look like and information about odour, which all service users were unaware of. Many participants believed antibiotics were necessary for wound healing and they believed that including information on the basic principles of antimicrobial resistance (AMR) would also be valuable.

*“I would have really liked if something is like inflamed, surrounding the area, that you know, that's most likely a sign of infection, that's what I thought. But also about like the swelling of the wound and the weeping of the wound, whether that's normal or not, because to me that wasn't particularly normal. I knew that you'd probably get swelling around the site, but not the weeping of it.” (Participant with necrotic wound)*

*“Another point is smell. My wound began to smell, and it wasn't until I saw the Consultant, that it was fully explained to me.” (Participant with necrotic wound)*

### Theme 3: skin care advice and healthy lifestyle behaviours

Healthy lifestyle habits are vital in wound care education, supporting a holistic approach for both service users and HCPs. Advice on skin care, moisturising, showering, avoiding smoking, proper nutrition, exercise, and the effects of specific medications on healing was considered valuable to support shared care and was considered essential for inclusion in an educational resource.

*“If there was just something that explained if you were on certain medications, rather than be contacting a health care professional, blocking the phone lines to a GP or something like that because you were worried, if there was something like that, then it would take the worry away really.” (Participant with chronic wound)*

‘*I always get dry and itchy lower legs in February/March time, which I put down to the central heating. I use E45 cream to moisturise and ease the itching. One of the leaflets advised spreading the cream in a downward direction to avoid blocking hair follicles. Never heard of that before!’ (Participant with experience of diabetic wound)*

### Theme 4: format and accessibility

A leaflet was identified as the most accessible and effective way to provide shared wound care education, offering essential information without being overwhelming. Participants stressed the need for clear content and images covering wounds, wound care techniques, wound healing, infection signs, and dressing practices. While HCPs were uncertain about patient use, service users valued the leaflet for initial guidance and reference. A website was also seen as useful for more detailed information.

*“it's difficult to work out how much people might be affected by actual pictures of wounds so if it's more like you said, like a cartoon type thing, it's not too specific to a person. Its more impersonal isn't it, but its relative, but impersonal. Whereas an actual photograph of somebody's wound is more personal.” (Participant with experience of a non-healing surgical wound)*

*“To probably be given a leaflet, but then have the option to go to a website. But there are a lot of elderly people that aren't tech savvy and a lot of elderly people live on their own, or they're a couple whose children don't live near and don't use computers particularly, or if they do, not very much, just basic things and they might not want to do that. So, I think if you've got the option of what could be offered.” (Participant with experience of non-healing surgical wound)*

## Phase four: educational resource development

The final phase of the project employed an iterative, user-centred approach to refine the resource combining multiple modes of engagement to integrate clinical and service user perspectives. Informal focus groups with HCPs, and communications with service users through telephone discussions, email, text messaging via WhatsApp were undertaken to achieve consensus and ensure that the resources were appropriate for implementation within community-based wound care. The outcome was the content for a patient education leaflet to support shared wound care within community settings, enhancing patient engagement, autonomy, and continuity of care. The resources included the main components identified as being important to both HCPs and service users throughout the stages of the research. Education on the signs and symptoms of infection, the normal wound healing trajectory, the importance of wound dressings and performing safe dressing changes, healthy skin and lifestyle behaviours and AMR were identified as core components for inclusion to enable patients to safely participate in shared wound management. Although no differentiation was made regarding wound types or underlying pathologies, both HPCs and service users agreed that while particular dressings and treatments may differ according to clinical circumstances, the foundational principles of shared wound care education are universally relevant. The findings suggest that a standardised, adaptable educational framework can effectively support shared wound care across diverse patient populations within community settings.

## Discussion

This study revealed that both service users and HCPs identified recognising infection, wound dressing selection and an understanding of the normal healing process as fundamental shared care priorities, reinforcing existing evidence that targeted education improves wound care knowledge, promotes adherence, and enhances patient confidence (Blackburn & Ousey, 2023; Kapp & Santamaria, 2017). Persistent inaccuracies among service users, such as beliefs about the need for frequent dressing changes or the benefits of “airing” wounds, highlight ongoing educational gaps and the importance of a co-production approach to shared care education. By addressing these concepts, and promoting evidence-based wound care knowledge, this co-produced resource supports informed decision-making and shared responsibility in care.

Inadequate understanding of wound healing trajectories and dressing functions was a recurring issue among service users, echoing studies highlighting misconceptions around dressing changes and wound healing (Van Hecke et al., 2009; Weller et al., 2016). This aligns with previous research suggesting that insufficient knowledge of infection contributes to anxiety, overuse of antibiotics, and delays help seeking (Chitambira, 2019). Including information on AMR within wound education supports national priorities for antimicrobial stewardship (NWCSP, 2020; NICE, 2024) and may promote more judicious antibiotic use. Participants expressed a preference for receiving education in multiple formats, particularly printed leaflets complemented by online resources, reflecting the importance of multimodal learning in supporting accessibility. For patients with limited digital literacy, the leaflet format ensures inclusivity, while digital platforms offer opportunities for deeper engagement, consistency, and scalability (Moore et al., 2022). Table I presents the key elements of shared wound care education that should be considered when creating an educational resource.

**Table I.** Components of shared wound care education.

Component	Objective
Recognising signs and symptoms of infection and inflammation.	Support early intervention.
	Reduce service user anxiety and promote safety and confidence in shared care.
	Reduce unnecessary antibiotic use through better monitoring.
	Support collaborative wound management between patients and professionals.
Wound dressing and mechanisms of wound healing	To improve understanding of wound healing and promote self-management.
	Reduce gaps in knowledge and promote appropriate wound care.
	Ensures service users carry out safe and consistent wound management, supporting safe shared care.
AMR	Promote responsible use of antibiotics.
Skin care advice and healthy lifestyle behaviours	Accelerate healing and maintain healthy skin.
	Prevent recurrence and secondary complications.
	Empower patients to take ownership of their care, improving long-term outcomes and reducing reliance on healthcare services.
Format considerations	Support multimodal learning, accessibility and inclusivity.

This study contributes to a growing body of evidence supporting co-production in health education, demonstrating that shared development between service users and clinicians can produce resources that are more relevant, usable, and acceptable (Makey et al., 2022). As shared care necessitates and understanding of active involvement (Clemett et al., 2024), co-production ensures that educational materials are accessible, evidence-based, and reflect lived experiences, whilst acknowledging contextual barriers such as health literacy and digital exclusion (NHS England, 2019; Moore & Coggins, 2021), and ensures patients are active partners in care rather than passive recipients. The findings reinforce the role of shared wound care in enhancing patient autonomy and continuity of care and supports previous findings demonstrating how engaging patients in wound care activities is linked to improved healing outcomes, reduced clinical workload, and increased satisfaction (Clemett et al., 2024; Žulec et al., 2019).

## Limitations

The research involved a small, purposive sample and the participants may have higher engagement or health literacy than the wider wound care population. Both service users and HCP participants were self-selecting, potentially reflecting those with greater motivation, engagement, or positive attitudes toward shared care and education. Despite these limitations, the study provides valuable exploratory evidence to guide the development and evaluation of shared wound care education for community nursing practice to the inform future development of shared wound care education.

## Conclusions

By integrating the perspectives of both service users and HCPs, this project identified key educational priorities, delivery preferences, and contextual factors influencing patient engagement in shared wound care. The findings highlight the importance of education that is co-designed and person-centred, ensuring that patients are empowered to participate in their own care, and demonstrates the importance of a sequential co-design approach to patient engagement. Implementing shared wound care education as part of routine practice may also advance greater patient engagement and satisfaction, supporting the broader shift towards partnership-based care. Future research should examine the implementation and effectiveness of this framework in practice, including the impact on patient knowledge, confidence, and healing outcomes, exploring its scalability, sustainability, and long-term benefits for both patients and healthcare systems. Additionally, exploring HCPs' perspectives on delivering shared wound care education would provide valuable insights into embedding co-production in practice.

## Data Availability

Data will be made available on reasonable request.

## Source(s) of support

An educational grant from Essity BSN Medical facilitated the conduct of the work described in the article.
